# Socioeconomic drivers of human Brucellosis in Ningxia, China: A one health and spatiotemporal analysis for targeted intervention

**DOI:** 10.1371/journal.pntd.0014124

**Published:** 2026-03-16

**Authors:** Ping Zhang, Xiaojuan Ma, Ting Pan, Jingxia Dang, Dongfeng Pan, Mingbo Chen, Peifeng Liang

**Affiliations:** 1 School of Public Health, Ningxia Medical University, Yinchuan, China; 2 Ningxia Key Laboratory of Environmental Factors and Chronic Disease Control, Yinchuan, China; 3 Department of Emergency Medicine, People’s Hospital of Ningxia Hui Autonomous Region, Ningxia Medical University, Yinchuan, China; 4 Department of Medical Affairs, People’s Hospital of Ningxia Hui Autonomous Region, Ningxia Medical University, Yinchuan, China; Makerere University, UGANDA

## Abstract

**Objective:**

This study aimed to investigate the spatiotemporal heterogeneity of human brucellosis and quantify the exposure-lag-response relationships of key socioeconomic and livestock production drivers in Ningxia, China, from 2007 to 2022. The goal was to generate evidence for developing targeted, integrated interventions in this high-burden pastoral region.

**Methods:**

We conducted a retrospective ecological study integrating human brucellosis surveillance data with county-level socioeconomic and livestock production statistics. A multi-analytic framework was employed: Joinpoint regression analyzed long-term trends; spatiotemporal scan statistics identified high-risk clusters; GeoDetector quantified the explanatory power of potential drivers on spatial heterogeneity; and Distributed Lag Nonlinear Models (DLNMs) were constructed to assess the nonlinear and lagged effects of significant drivers on monthly incidence.

**Results:**

The human brucellosis incidence rate in Ningxia increased 167-fold, from 0.52 to 86.83 per 100,000 population between 2007 and 2022. Spatiotemporal analysis revealed a persistent high-risk cluster (Relative Risk, RR = 4.22, P < 0.001) in 11 eastern counties. GeoDetector identified livestock-related factors as primary spatial drivers, with sheep inventory (q = 0.96) and cattle inventory (q = 0.92) showing the highest explanatory power. DLNM results indicated a significant 3-year lagged risk associated with low cattle stocking levels (RR = 2.75), while sheep stocking exhibited a complex, non-linear U-shaped lag effect. In contrast, higher regional Gross Domestic Product (GDP) was associated with an immediate lower risk (RR = 0.81).

**Conclusion:**

The brucellosis epidemic in Ningxia is characterized by intense spatial clustering and is associated with distinct, lagged effects of livestock production structures coupled with immediate economic influences. The findings underscore that livestock production metrics can serve as effective proxies for risk mapping even in the absence of direct animal infection data. Our study highlights the necessity for a dual-strategy intervention: implementing risk-based veterinary public health measures in high-incidence clusters while leveraging economic development to strengthen long-term prevention and control capacities.

## Author summary

Brucellosis is a zoonotic disease that spreads from animals to humans, posing a significant public health threat, especially in pastoral regions like Ningxia, China. Understanding the complex drivers behind its resurgence is critical for designing effective control measures. In this study, we analyzed human brucellosis cases in Ningxia from 2007 to 2022 using an integrated approach that combines spatial mapping, statistical detection of driving factors, and time-lag modeling. We found that the epidemic intensified dramatically and became concentrated in eastern counties. The number of sheep and cattle was the strongest factor determining where cases occurred. Our models revealed that risks operate on different timelines: higher economic development was immediately associated with lower risk, likely by enabling better prevention. In contrast, a higher risk was observed three years after an increase in cattle numbers in low-stock areas, suggesting a delayed spillover from smallholder farms where infections may go undetected. This lag provides a crucial window for early intervention. Our study demonstrates that routinely collected livestock and economic data can serve as powerful proxies to map disease risk and forecast outbreaks, even in the absence of comprehensive animal testing. The results argue for targeted, dual-track strategies: intensifying animal vaccination in high-risk clusters while using economic incentives to improve farm biosecurity and launching early warnings based on livestock trends to prevent future surges.

## Introduction

Brucellosis remains a major global zoonotic disease, with transmission sustained at the human-animal interface in over 170 countries and territories [1]. The annual incidence is estimated at 1.6 to 2.1 million cases, with Asia bearing the largest burden of approximately 1.2 to 1.6 million new cases each year [1]. In China, concerted national control efforts successfully suppressed human and animal brucellosis to low levels following severe epidemics before the 1980s [2]. However, a dramatic resurgence began in the mid-1990s. By 2021, China reported 73,645 human cases, with the disease ranking as the fifth most common notifiable infectious disease nationally—a stark rise from its 16th place ranking in 2000 [3,4]. This reversal underscores the complex and persistent challenges in controlling brucellosis within evolving socioeconomic and agricultural landscapes.

The transmission dynamics of brucellosis are governed by a multifactorial system. Beyond the essential biological components—animal reservoirs (primarily cattle, sheep, and goats), human hosts, and environmental persistence of the pathogen—a constellation of ecological and anthropogenic drivers modulates risk [5]. These include climatic variables (e.g., temperature, rainfall), land use, and critically, human behaviors and husbandry practices such as feeding methods, hygiene during animal contact, and consumption of unpasteurized dairy products [6–8]. The synergy between livestock production intensification, economic activities, and sometimes limited veterinary public health capacity can fuel the geographic expansion of infected areas [6,9]. Consequently, effective and sustainable control strategies require a holistic, system-level understanding that integrates these human, animal, and environmental dimensions—a core tenet of the One Health approach [10].

Despite growing recognition of this complexity, many epidemiological studies on human brucellosis remain primarily descriptive, focusing on one-dimensional analyses of temporal trends or static spatial patterns [11]. This approach often fails to adequately capture the interacting spatiotemporal heterogeneities of driving factors, leading to fragmented and sometimes inconsistent evidence for planning interventions [6]. There is a pressing need for analytical frameworks that can simultaneously identify high-risk areas, disentangle the individual and interactive contributions of key drivers, and quantify the dynamic, time-lagged effects these factors have on disease incidence. Such an integrated analysis is crucial for moving beyond correlative insights towards understanding mechanistic pathways and identifying precise levers for intervention.

The Ningxia Hui Autonomous Region in northwestern China epitomizes these challenges and represents an extreme case within the national epidemic. As a major pastoral region where animal husbandry contributes roughly 30% of agricultural output, livestock is a vital economic pillar [12]. Historically a low-incidence area, Ningxia has experienced an explosive increase in human brucellosis since 2008. By 2021, its reported incidence rate reached 73.50 cases per 100,000 population—approximately 14 times the national average and the highest among all Chinese provinces [3,13]. This surge has coincided with rapid, policy-driven expansion of cattle and sheep production [14], yet the precise mechanisms linking livestock sector dynamics, socioeconomic conditions, and the spatiotemporal pattern of human disease remain inadequately elucidated. Prior local studies have confirmed correlations between livestock numbers and human cases but have not fully explored the nonlinear and lagged relationships within a unified analytical framework that accounts for both spatial clustering and temporal evolution [8,14].

### Conceptual framework and analytical approach

Guided by the One Health principle, our analysis is structured around a conceptual framework that links distal socioeconomic drivers, proximal livestock production factors, and human brucellosis outcomes through potential environmental and behavioral exposure pathways. This framework acknowledges the inherent spatiotemporal lags in zoonotic transmission. To operationalize it, we employ an integrated suite of methods, each interrogating a specific component of the system: (1) Spatiotemporal scan statistics identify where and when human case outcomes cluster, revealing the footprint of the epidemic. (2) The GeoDetector method quantifies the extent to which the spatial stratification of candidate socioeconomic and livestock production drivers explains this observed heterogeneity, identifying key static determinants. (3) Distributed Lag Nonlinear Models (DLNMs) decode the dynamic, often delayed effects of these key drivers on monthly incidence, capturing the temporal dimension of risk propagation from the animal reservoir to human populations. This sequential application—from pattern detection, to spatial determinant quantification, to temporal dynamic modeling—provides a mechanistic understanding of the epidemic system, moving beyond correlation to inform targeted intervention.

To address this critical knowledge gap, we conducted a comprehensive ecological study covering the period 2007–2022 with three specific objectives... By employing an integrated suite of analytical techniques, we aim to unveil the complex mechanisms underlying the epidemic’s intensification. Conceptually, our study is guided by a systems framework that links upstream socioeconomic and livestock production drivers to human disease outcomes through exposure pathways at the human-animal interface. Our analytical approach directly interrogates this framework: (1) spatiotemporal scan statistics identify where and when high-risk human outcomes cluster; (2) the geographic detector method quantifies the extent to which upstream drivers explain this spatial heterogeneity; and (3) distributed lag nonlinear models decode the dynamic, often delayed effects of these drivers on incidence, reflecting the lagged nature of transmission through animal reservoirs and exposure pathways. The findings are intended to provide a robust, evidence-based foundation for designing targeted, spatially explicit, and temporally informed prevention and control strategies, thereby contributing to the broader application of One Health principles in pastoral regions burdened by zoonotic diseases.

## Materials and methods

### Ethics statement

The study was based on publicly available epidemiologic data and statistical yearbook information and did not involve human subjects or animal experimentation, so ethical approval was not required. The study protocol was approved by the Ethics Review Board of the Ningxia Centers for Disease Control and Prevention (NXCDC_IRB2023_11).

### Study design and data sources

We conducted a retrospective ecological study covering all counties in the Ningxia Hui Autonomous Region from 2007 to 2022. Human brucellosis surveillance data were extracted from the Ningxia Center for Disease Control and Prevention’s (CDC) Notifiable Disease Surveillance System. All cases were diagnosed according to the unified national criteria and mandatorily reported within 24 hours. The anonymized dataset included geolocation (township), basic demographics, and high-risk occupation codes (e.g., farmer, herdsman, veterinarian). Based on these codes, cases were classified into broad occupational categories (predominantly farmer/herdsman, with others including veterinarian and slaughterhouse worker). Throughout the study period, over 85% of reported cases were classified as farmers or herdsmen, confirming the predominance of occupational exposure in this pastoral setting. However, due to the ecological study design and privacy protections, individual-level behavioral data (e.g., specific husbandry practices, raw milk consumption) were not available for analysis. To address a key data limitation common in pastoral settings—the frequent lack of integrated, county-level animal disease surveillance—we utilized livestock production and economic statistics as proxy indicators for transmission risk. These proxy data, including annual livestock inventory (cattle, sheep), production outputs (beef, mutton, dairy), gross output value of animal husbandry, and per capita Gross Domestic Product (GDP), were obtained from the official Ningxia Statistical Yearbook for the corresponding years. All data were harmonized at the county level, de-identified, and are publicly archived in Dryad (DOI: [Reserved]), with township-level coordinates generalized to protect privacy.

### Analytical framework and statistical methods

A multi-stage analytical framework was implemented to first describe patterns, then identify spatial drivers, and finally quantify dynamic exposure-lag-response relationships. All analyses were performed using R software (version 4.3.0), with spatial mapping conducted in ArcGIS Pro (version 10.8).

#### 1. Spatiotemporal distribution and cluster analysis.

To characterize the epidemic’s evolution, we employed a combination of descriptive and model-based techniques. The geographic progression of incidence was visualized using sequential choropleth maps. Temporal trends were analyzed using Joinpoint regression (Joinpoint Regression Program, version 5.0.2) to identify significant inflection points in the annual incidence rate series [15,16]. The Annual Percentage Change (APC) for each segment and the Average Annual Percentage Change (AAPC) for the entire period were calculated to quantify the pace of change. To detect areas with unusually high incidence in space and time, a retrospective space-time scan statistic was performed using SaTScan software (version 10.2.5) [17]. A discrete Poisson model was used, with the county resident population as the background. The analysis scanned for clusters of high rates using a circular window, and statistical significance was evaluated with 999 Monte Carlo permutations (P < 0.05).

#### 2. Identification of spatial determinants.

To move beyond correlation and identify factors responsible for the observed spatial heterogeneity, we conducted a two-step analysis. First, Spearman‘s rank correlation was used for an initial screening of monotonic associations between annual incidence and all candidate socioeconomic and livestock variables [18]. Subsequently, the GeoDetector (GeoDetector) method was applied to quantify the explanatory power (q-statistic, ranging from 0 to 1) of each factor’s spatial stratification on the disease’s spatial distribution [2,13]. This method is robust for detecting spatial associations in ecological studies. Continuous variables were discretized using an optimal classification algorithm to minimize subjective bias. Furthermore, the interaction detector module was used to assess whether paired factors acted independently or exhibited nonlinear, synergistic effects in driving spatial risk patterns.

#### 3. Modeling lagged and nonlinear exposure-response relationships.

To address the temporal dimension of risk and quantify the often-overlooked delayed effects of drivers, we constructed a Distributed Lag Nonlinear Model (DLNM) [19]. This approach models the potentially nonlinear relationship between an exposure and an outcome across a pre-defined lag period. Key predictors, primarily those identified with high q-values by the GeoDetector, were standardized (Z-score) and entered into the model. Prior to final model fitting, variable selection was performed using the Least Absolute Shrinkage and Selection Operator (LASSO) regression to prevent overfitting and identify the most parsimonious set of predictors from the candidate pool [20]. The core of the DLNM is a cross-basis function, which simultaneously describes the exposure-response curve and the lag-response curve. We specified a natural cubic spline with three degrees of freedom for both the exposure and lag dimensions, with a maximum lag of three years to capture mid-term effects. The model was embedded within a negative binomial regression framework to account for over-dispersion in the monthly case count data. Model fit was evaluated using the pseudo R², Akaike Information Criterion (AIC), and Bayesian Information Criterion (BIC). Standard residual diagnostics were conducted to ensure model assumptions were met (see Supplementary Material).

## Results

### 1. Spatiotemporal evolution of the brucellosis epidemic

**Fig 1:**
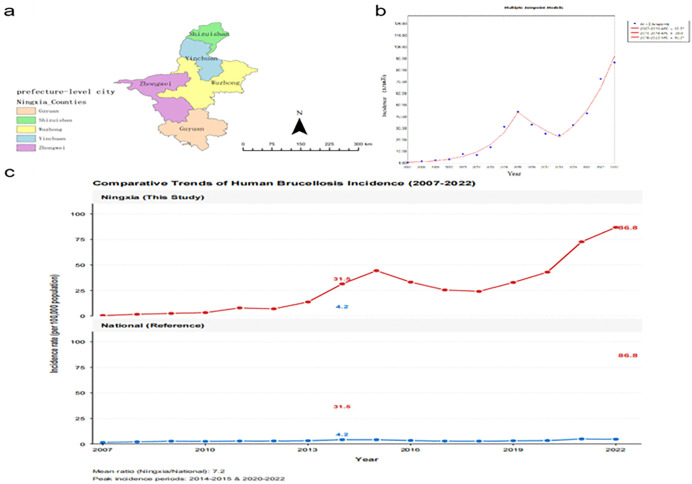
Temporal distribution of human brucellosis in Ningxia and China (2007–2022). (a) Administrative divisions of Ningxia Hui Autonomous Region, China. Map (a) shows the study area and its internal boundaries of five prefecture-level cities in Ningxia. The base map layer was created using data from the Database of Global Administrative Areas (GADM, https://gadm.org), which is available under a Creative Commons Attribution 4.0 International (CC BY 4.0) license.; (b) Joinpoint trend analysis of brucellosis incidence in Ningxia; (c) Comparative Trends of Human Brucellosis Incidence (2007-2022).

The study was conducted in the Ningxia Hui Autonomous Region, a major pastoral area in northwestern China, with its internal administrative divisions into five prefecture-level cities shown in Fig 1a. Between 2007 and 2022, the region experienced a dramatic intensification of the human brucellosis epidemic. The overall incidence rate surged from 0.52 to 86.83 per 100,000 population, a 167-fold increase. Notably, the incidence in Ningxia consistently and substantially exceeded the national average throughout the latter part of the study period, with the divergence becoming particularly pronounced after 2015 (Fig 1c).

**Fig 2:**
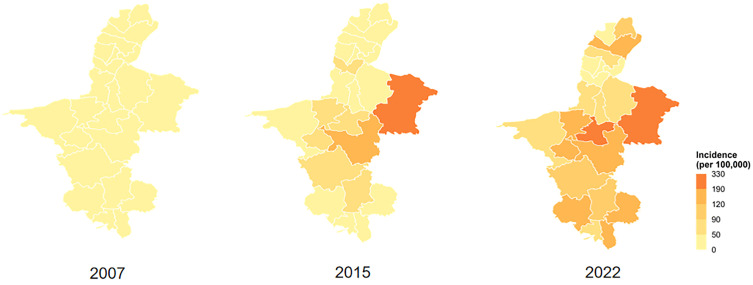
Temporal-spatial distribution of brucellosis incidence in Ningxia (2007–2022). County-level annual incidence rates (per 100,000 population) are categorized into five levels: 0.00–50.00, 50.01–90.00, 90.01–120.00, 120.01–190.00, and 190.01–330.00. Administrative boundaries were obtained from the ‘geoBoundaries-CHN-ADM3_simplified.geojson’ dataset on the Humanitarian Data Exchange (HDX) platform, available directly at: https://data.humdata.org/dataset/geoboundaries-admin-boundaries-for-china/resource/bb2bb8b4-c882-4eb0-93e8-f0ed0e26b740 under a Creative Commons Attribution 4.0 International (CC BY 4.0) license (https://creativecommons.org/licenses/by/4.0/). The map is for illustrative purposes only and does not imply any opinion on legal status.

Joinpoint regression analysis of the temporal trend delineated three distinct phases: a period of rapid increase from 2007 to 2015, a brief interim decline from 2015 to 2018, and a subsequent resurgent rise from 2018 to 2022 (Fig 1b). Spatially, the epidemic underwent a profound transformation. It evolved from a state of low and relatively homogeneous incidence across the region in 2007 to the emergence of a discernible eastern core by 2015. This core subsequently expanded and consolidated, forming a well-defined, contiguous high-risk zone encompassing 11 counties and districts in the central and eastern parts of Ningxia by 2022 (Fig 2), (the complete annual series is provided in S2 Fig).

Space-time scan analysis confirmed the presence of statistically significant spatiotemporal clusters (P < 0.001, Table 1). The most prominent was a persistent, high-risk cluster (Cluster 1), active from January 2015 to December 2022. This cluster covered 11 counties in central and eastern Ningxia and was associated with a 4.22-fold higher relative risk (RR) compared to areas outside the cluster. Four additional, transient clusters of lower intensity and shorter duration were identified in other parts of the region (Clusters 2–5, Table 1).

**Table 1:** Results of Spatio-Temporal Scan Analysis of Ningxia Region, 2007-2022.

Cluster	Region	Coordinates/ radius	Time frame	Relative Risk (RR)	Log likelihood ratio (LLR)		*P*-value
Cluster1	Jingyuan,Longde,Panyang,Yuanzhou District,Xiji,Haiyuan,Tongxin,Hongshipu,Zhongning,Shapotou,Yanchi	(35.52N,106.34 E)/ 242.62 km	2015/1/1–2022/12/31	4.22		7368.80	< 0.001
Cluster2	Dawukou,Pingluo, Huinong, Helan	(39.08N,106.33 E)/ 44.75 km	2021/1/1–2022/12/31	2.77		603.64	< 0.001
Cluster3	Hongshipu	(37.37N,106.21 E)/ 0 km	2009/1/1–2013/12/31	2.3		142.53	< 0.001
Cluster4	Yanchi	(37.62N,107.06 E)/ 0 km	2011/1/1–2013/12/31	2.63		113.82	< 0.001
Cluster5	Yongning	(38.30N,106.09 E)/ 0 km	2015/1/1–2015/12/31	2.12		30.80	< 0.001

Joinpoint analysis of incidence trends within these clusters revealed heterogeneous temporal dynamics (Table 2). The core, persistent Cluster 1 exhibited an initial phase of explosive growth from 2007-2009 (Annual Percentage Change, APC = 484.2%), followed by a prolonged period of sustained, albeit slower, transmission from 2009-2022 (APC = 20.9%). The transient clusters (Clusters 2–5) showed more variable temporal patterns, including phases of rapid growth, decline, or stability, as detailed in Table 2.

**Table 2:** Joinpoint analysis of brucellosis incidence in notable agglomerations, 2007-2022.

Cluster	Endpoint	APC(95%CI)	AAPC(95%CI)
Cluster1	2007 ~ 2009	484.2*(62.3 ~ 2003.1)	49.1*(27 ~ 75.1)
	2009 ~ 2022	20.9*(13 ~ 29.3)	
Cluster2	2007 ~ 2010	-11.5(-50.8 ~ 59.1)	33*(12 ~ 59)
	2010 ~ 2014	100.5*(11.5 ~ 260.4)	
	2014 ~ 2022	26.9*(11.7 ~ 44.3)	
Cluster3	2007 ~ 2009	779.8*(176.3 ~ 2701.3)	44.6*(22.3 ~ 71.1)
	2009 ~ 2018	-9.9(-20.6 ~ 2.2)	
	2018 ~ 2022	70.3(18.1 ~ 145.6)	
Cluster4	2007 ~ 2014	102.5*(57.8 ~ 159.9)	39.7*(21.2 ~ 60.9)
	2014 ~ 2022	0.8912(-17.7 ~ 23.7)	
Cluster5	2007 ~ 2015	56.6*(29.4 ~ 89.6)	29.8*(13.7 ~ 48.3)
	2015 ~ 2022	4.8(-17.1 ~ 32.4)	

### 2. Socioeconomic and livestock production drivers of spatial heterogeneity

**Fig 3:**
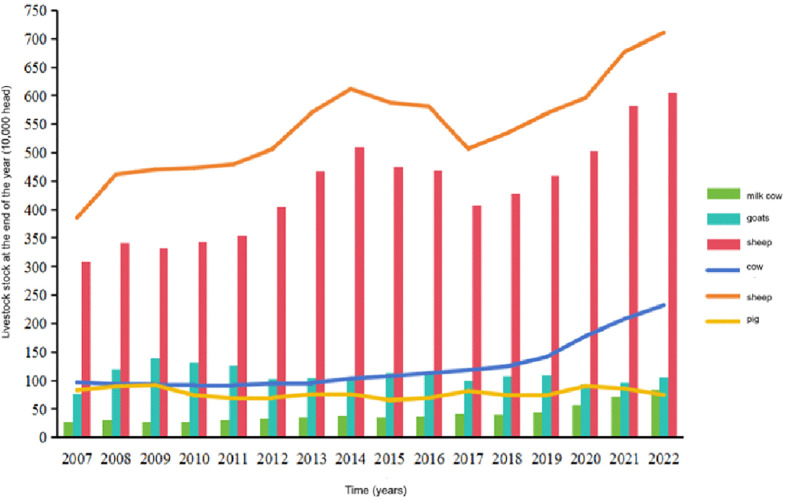
Livestock stock at the end of the year, Ningxia region, 2007-2022.

To identify the factors underlying the observed spatial patterns, we analyzed the role of key socioeconomic and livestock production variables. The livestock inventory structure in Ningxia during the study period was overwhelmingly dominated by sheep, which consistently constituted over 95% of the total stock and showed a marked upward trend, peaking in 2022. In contrast, cattle, goat, and pig inventories remained relatively stable (Fig 3).

**Fig 4:**
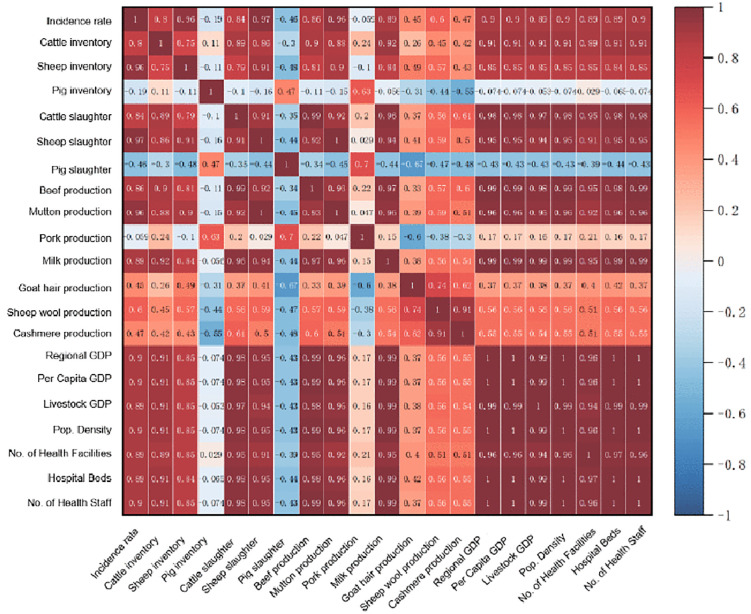
Spearman’s correlation coefficient between socio-economic factors and the incidence of brucellosis in Ningxia region, 2007-2022.

Initial screening using Spearman correlation analysis revealed significant positive monotonic associations (P < 0.05) between annual brucellosis incidence and 16 indicators, including cattle and sheep inventories, meat and dairy production, gross livestock output value, and regional GDP. Notably, no significant correlation was found with swine-related indicators, underscoring the central role of cattle and sheep husbandry alongside the regional economy in the epidemic (Fig 4).

We then applied the GeoDetector method to quantify the explanatory power of each factor for the observed spatial heterogeneity in incidence. The analysis identified several factors with exceptional explanatory power (q-statistic > 0.90). Sheep inventory (q = 0.963) and regional GDP (q = 0.958) emerged as the strongest individual determinants, followed closely by cattle inventory (q = 0.924) and the gross output value of animal husbandry (q = 0.936) (Table 3). This identifies the scale and economic output of ruminant (cattle and sheep) husbandry as the primary factors associated with the disease’s spatial distribution.

**Table 3:** Factor detector results.

Factor	*q* statistic	*p* value
X1(number of cattle)	0.9237	0.0058
X2(number of sheep stocked)	0.9629	0.0000
X3(number of cows slaughtered)	0.5689	0.1026
X4(number of sheep slaughtered)	0.9574	0.0000
X5(Beef production)	0.9489	0.0000
X6(Lamb production)	0.9574	0.0000
X7(Milk production)	0.9581	0.0000
X8(Goat wool production)	0.3110	0.8909
X9(Sheep wool production)	0.6152	0.3304
X10(Cashmere production)	0.4312	0.4564
X11(Gross regional product)	0.9584	0.0000
X12(Gross regional product per capita)	0.9584	0.0000
X13(Gross value of livestock production)	0.9358	0.0032
X14(Population density)	0.9584	0.0000
X15(Number of medical and health institutions)	0.8444	0.0171
X16(Number of beds in medical and health institutions)	0.8554	0.0000
X17(Number of health personnel)	0.9584	0.0000

**Fig 5:**
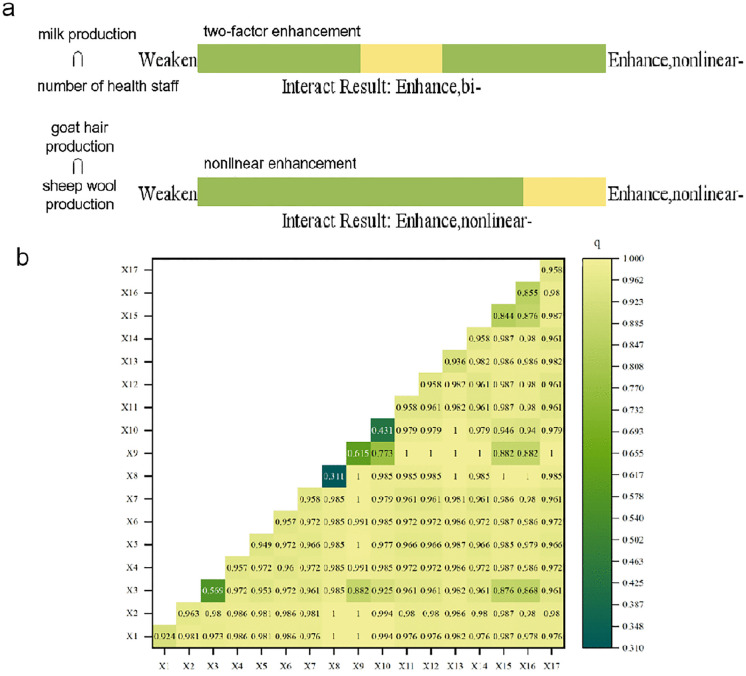
Two-factor enhanced interactions driving brucellosis in Ningxia. (a) Factor pairs and interaction patterns; (b) Quantified interaction *q*-values.

Interaction detector analysis further revealed that the combined influence of two factors often resulted in a nonlinear, enhanced effect greater than the sum of their individual impacts (Fig 5). Key driving factors consistently exhibited interactive explanatory power exceeding 80%, underscoring that the spatial risk pattern was driven by the complex interplay between livestock production intensity and socioeconomic conditions.

### 3. Lagged and nonlinear effects of key drivers on incidence risk

To elucidate the dynamic, temporal relationship between key drivers and disease risk, we constructed a Distributed Lag Nonlinear Model (DLNM). Variable selection prior to fitting was performed using LASSO regression, refining the predictor set from the factors highlighted in the GeoDetector analysis.

The final DLNM revealed distinct temporal patterns of association (Table 4). A significant delayed effect was observed for standardized cattle inventory. Specifically, an increase in cattle inventory was associated with a peak in relative risk (RR) at a three-year lag (RR = 2.75, 95% CI: 1.22–6.18). For standardized sheep inventory, an increase was associated with a reduced risk at both one-year and three-year lags (RR = 0.27 and 0.08, respectively). In contrast, several factors showed immediate (lag 0) effects: increases in beef and dairy production were associated with elevated contemporaneous risk (RR = 1.25 and 1.34, respectively), whereas an increase in regional GDP exhibited a protective effect (RR = 0.81). The model confirmed the exceptionally high baseline risk in the identified spatiotemporal clusters, with Yanchi and Hongsipu counties having RRs of 38.0 and 27.6, respectively, and captured a strong temporal trend of exponentially escalating risk from 2007 to 2022 (Table 4).

**Table 4:** Distributed lag nonlinear model (DLNM) regression results.

variable	lag order (math)	RR	95% CI	*p*-value
Cattle stock(Z)	l3	2.75**	1.22–6.18	0.012
Sheep stock(Z)	l1	0.27*	0.07–0.99	0.027
	l3	0.08***	0.02–0.36	<0.001
beef production(Z)	imminent	1.25*	1.02–1.53	0.037
mutton production(Z)	imminent	1.16	0.88-1.53	0.3
dairy production(Z)	imminent	1.34***	1.13–1.61	0.004
Region GDP(Z)	imminent	0.81*	0.68–0.97	0.02
High-risk counties	–	–	–	–
Yanchi	–	38.0***	13.6–107	<0.001
Hongsipu	–	27.6***	13.1–58.5	<0.001
Temporal Effects	–	–	–	–
2007 (Reference)	–	1	–	–
2013	–	45.6***	24.9–86.2	<0.001
2020	–	583***	273–1,271	<0.001
2022	–	1,639***	659–4,160	<0.001

**Fig 6:**
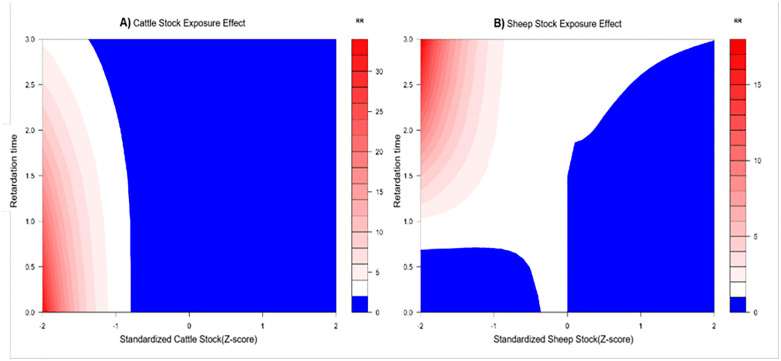
Results of a Distributed Lag Nonlinear Model Analyzing the Exposure Effect of Cattle and Sheep Stocking: Changes in Relative Risk (RR) with Standardized Stocking and Lag Time.

**Fig 7:**
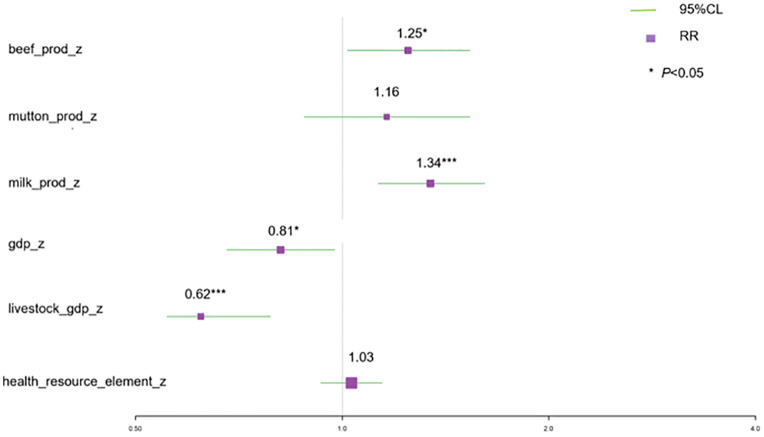
Relative Risk (RR) and 95% Confidence Interval for Immediate Effects of Covariates.

Visualization of the exposure-lag-response relationships detailed these complex, nonlinear dynamics (Fig 6). For cattle inventory, elevated risk was concentrated at longer lags (2–3 years) specifically when inventory levels were below the regional average (Z-score < 0) (Fig 6A). For sheep inventory, a “U-shaped” risk pattern emerged across the lag dimension, where both extremely low and high standardized inventory levels were associated with increased risk at specific lag periods (Fig 6B). The direction and magnitude of the immediate effects for other significant covariates are summarized in Fig 7.

## Discussion

This study provides a comprehensive integrated spatiotemporal analysis of the severe and escalating epidemic of human brucellosis in Ningxia, China, from 2007 to 2022. By employing a sequential analytical framework, we have moved beyond descriptive epidemiology to delineate the systemic drivers and dynamic mechanisms underlying this profound public health challenge. Our core finding is that the epidemic’s intensity and persistence are fundamentally orchestrated by specific, time-lagged interactions between livestock production and socioeconomic factors, crystallizing a critical “One Health” dilemma in pastoral development. Our modeling results translate this abstract concept into quantifiable mechanisms, revealing not only what drives the epidemic but how and when risks manifest across the human-animal interface. Beyond these specific findings for Ningxia, our study demonstrates the broader utility of the integrated analytical framework itself—sequencing spatiotemporal clustering, spatial determinant detection, and temporal lag modeling—to unravel systemic drivers of zoonotic epidemics where direct animal infection data are scarce. This approach operationalizes the One Health principle by quantitatively linking livestock sector dynamics and socioeconomic conditions to human disease patterns across space and time, providing a replicable model for resource-limited settings.

### 1. Reinterpreting spatiotemporal patterns through a systems lens

The evolution from a low, homogeneous incidence to a tightly clustered epidemic in eastern Ningxia is a signature of a system under stress. The identification of a persistent, high-risk core cluster (RR = 4.22, 2015–2022) aligns precisely with regions that have undergone intensive specialization in ruminant husbandry [14]. This spatial convergence of high livestock density and high human disease burden mirrors patterns in other pastoral provinces [6], suggesting a common developmental pathway where agricultural economic growth outpaces integrated risk management capacity. The temporal trajectory—characterized by explosive initial growth followed by sustained endemic transmission—reflects an initial phase of rapid livestock sector expansion and the subsequent establishment of resilient transmission cycles. This pattern underscores that brucellosis control in such settings must be addressed as a systemic consequence of production intensification, not merely as an infectious disease outbreak.

### 2. Unpacking the mechanistic drivers: scale, synergy, and lagged effects

Our application of GeoDetector and DLNM models allowed us to decompose the drivers into static spatial forces and dynamic temporal processes. The quantification of exceptional explanatory power (q > 0.92) for sheep and cattle inventory formally confirms that the scale of the ruminant reservoir is the paramount determinant of geographic risk [8,13]. More importantly, the interaction analysis revealed that this risk is nonlinearly amplified when high livestock intensity coincides with other vulnerabilities, such as limited healthcare access (e.g., interaction q increasing from 0.92 to 0.98). This quantifies a key “One Health” tenet: risks originating in the animal sector are magnified by constraints in the human health sector.

The DLNM findings provide crucial insights into the timing of risk, revealing temporal patterns consistent with cross-sectoral dynamics. The significant association of higher regional GDP with lower risk (RR = 0.81 at lag 0) is consistent with studies linking economic development to immediate improvements in preventive capacities, such as investment in animal vaccination programs or personal protective equipment [3,8]. In stark contrast, the “low-stock, long-lag” risk pattern associated with cattle (peak RR = 2.75 at a 3-year lag) is mechanistically revealing. This finding is particularly insightful. In the context of Ningxia’s mixed farming systems, it may reflect a smallholder risk profile [21]. In these settings, limited resources can delay investments in biosecurity, veterinary care, and testing. An initially introduced or endemic low-level infection in a herd may thus smolder subclinically, with management deficiencies allowing for gradual pathogen amplification. The eventual spillover [22] to humans, often after several production cycles, manifests as this observed multi-year lag. It likely indicates scenarios in smallholder systems where subclinical infection in herds smolders for years before eventual spillover to humans, representing a delayed but potent risk from the animal health domain [14,23].

The immediate association of increased dairy production with higher risk (RR = 1.34 at lag 0) points to high-exposure value chains at the human-animal interface, such as unhygienic milking or processing of raw milk products [9]. Conversely, the complex “U-shaped” lag effect for sheep suggests risks at both extremes of management intensity. Collectively, these temporal patterns highlight the need for interventions that are specifically tailored to the different risk profiles of cattle and sheep production systems.

### 3. Towards precision public health: Implications for intervention

Our findings argue for a shift from uniform policy to a dual-track, precision public health strategy informed by spatiotemporal mechanisms. First, in the persistent high-risk spatial cluster (RR = 4.22), where livestock density is the paramount driver, resources must be concentrated on preemptively targeting the animal reservoir. This warrants implementing mandatory, subsidized ruminant vaccination campaigns coupled with movement controls in these counties [11]. Second, the immediate association of economic development with lower risk (GDP RR = 0.81) suggests that financial policy can be leveraged to build sustainable biosecurity. Incentives such as tax breaks or insurance premium subsidies could be tied to farmers’ adoption of certified measures (e.g., segregated lambing areas, regular testing). Third, the 3-year lagged risk signal from cattle inventory changes provides a critical lead time for early warning. A composite risk index, integrating real-time livestock and economic data, could trigger proactive ‘alert phases’ in areas predicted to experience elevated risk 1–2 years later, enabling targeted awareness campaigns and market inspections [17]. Finally, the immediate risk linked to dairy production (RR = 1.34) calls for focused food safety interventions, including strengthened enforcement of pasteurization regulations and promotion of affordable, small-scale pasteurization technology for local processors.

These distinct risk signatures—the “low-stock, long-lag” pattern for cattle and the “U-shaped” relationship for sheep—call for differentiated intervention strategies. For areas characterized by the ‘low-stock-high-risk’ signature for cattle, which may reflect smallholder production with limited veterinary access, interventions could prioritize subsidized or mobile veterinary extension services, affordable point-of-care diagnostics, and targeted health education on safe animal handling practices. Conversely, in regions dominated by sheep husbandry, where the U-shaped relationship indicates risks at both low and high management intensities, a dual approach is needed. For extensive grazing systems (low-density), enhanced surveillance and farmer education on zoonotic risks are key. For intensive systems (high-density), enforcement of biosecurity protocols, regular environmental disinfection in concentrated feeding operations, and stringent occupational health measures for workers should be the focus. Collectively, these patterns depict a “livelihood-security trap”: the very strategies employed to secure income—increasing livestock numbers, intensifying production—can inadvertently architect the conditions for sustained disease transmission across sectors.

### 4. Limitations, data constraints, and future directions

This study has limitations that also point to future research directions. The primary constraint is the absence of concurrent, county-level animal serosurveillance data, a common challenge in resource-limited pastoral regions. Additionally, as an ecological study using aggregated data, our findings demonstrate spatial and temporal associations rather than establishing direct causal links at the individual level. Unmeasured confounding factors and the ecological fallacy should be considered when interpreting the results. Second, we lacked granular data on individual risk behaviors (e.g., specific husbandry or consumption practices), which could refine our understanding of exposure pathways. Third, county-level aggregation may mask local heterogeneity [15,24]. Third, our ecological analysis was conducted at the county-level and annual/monthly scale. While appropriate for regional policy planning, this scale may mask finer-scale heterogeneities within counties and more immediate temporal fluctuations. The Modifiable Areal Unit Problem (MAUP) implies that the strength of the detected spatial associations could vary with different zoning schemes. Future studies incorporating township-level data or individual movement patterns could provide even more precise targeting for interventions. However, our study precisely demonstrates a pragmatic analytical pathway to circumvent the core data gap. We show that routinely collected livestock production and socioeconomic statistics—which are widely and freely available in many settings—can serve as powerful proxies for mapping brucellosis risk and understanding epidemic drivers with high explanatory power (q > 0.92). This provides a viable model for conducting preliminary risk assessments and prioritizing intervention zones in other data-scarce regions [11]. Future research should build upon this proxy-based risk mapping by integrating targeted animal and human serosurveys [25], along with behavioral surveys, within identified high-risk clusters to directly calibrate spillover rates and validate transmission mechanisms.

### 5. Conclusion and global significance

In conclusion, this study elucidates how brucellosis has become entrenched in Ningxia through a synergistic combination of livestock sector intensification and lagging parallel investments in veterinary public health. The epidemic’s trajectory, defined by a high-risk cluster (RR = 4.22), strongly associated with livestock scale (q > 0.92), and modulated by lagged (RR = 2.75 at lag 3) and immediate (RR = 0.81 at lag 0) socioeconomic factors, is a canonical example of a “One Health” failure mode. Breaking this cycle requires moving beyond siloed disease control to orchestrated, cross-sectoral policies that consciously align economic development incentives with health security objectives. The analytical framework employed here provides a transferable blueprint for understanding and addressing similar neglected zoonoses in pastoral communities worldwide, underscoring the imperative for sustainable development to be founded on intentionally integrated human, animal, and environmental health strategies, This necessitates proactive, data-driven platforms for joint risk assessment by public health and veterinary authorities [26].

## Conclusions

In conclusion, this study transcends descriptive epidemiology by applying an integrated analytical framework to decode the spatiotemporal epidemic system of human brucellosis in Ningxia. We demonstrate that livestock production scale (q > 0.92) is the dominant spatial factor associated with risk, while temporal risk is modulated through distinct immediate and lagged pathways---immediate association of economic level with lower risk (GDP RR = 0.81) versus delayed associations (e.g., cattle inventory RR = 2.75 at a 3-year lag) with livestock sector changes. This mechanistic understanding reveals a ‘livelihood-security dilemma.’ Resolving it requires shifting from passive disease reporting to proactive, intelligence-led control. Our proxy-based, multi-method framework offers a transferable blueprint for other data-scarce pastoral regions grappling with zoonotic diseases, highlighting that sustainable development must be explicitly designed to harmonize economic aspirations with health security across the One Health spectrum.

## Supporting information

S1 Fig Diagnostic plots for the final Distributed Lag Nonlinear Model (DLNM). Four-panel diagnostic plot of the negative binomial DLNM residuals: (A) residuals versus fitted values, (B) Q-Q plot of residuals, (C) scale-location plot, and (D) residuals versus leverage values with Cook‘s distance contours. These plots were used to validate key model assumptions including homogeneity of variance, distributional adequacy, and to identify potential influential observations.
(TIF)

S2 Fig Annual spatiotemporal distribution of brucellosis incidence in Ningxia (2007–2022). Administrative boundaries were obtained from the ‘geoBoundaries-CHN-ADM3_simplified.geojson’ dataset, available directly at: https://data.humdata.org/dataset/geoboundaries-admin-boundaries-for-china/resource/bb2bb8b4-c882-4eb0-93e8-f0ed0e26b740. The dataset is part of the geoBoundaries project and is available under a Creative Commons Attribution 4.0 International (CC BY 4.0) license (https://creativecommons.org/licenses/by/4.0/). The map is for illustrative purposes only and does not imply any opinion on legal status. County-level annual incidence rates (per 100,000 population) for all study years from 2007 to 2022 are displayed using the same classification as in Fig 2. This comprehensive series illustrates the year-by-year evolution of high-incidence areas.
(TIF)
