# Correction: Socioeconomic drivers of human Brucellosis in Ningxia, China: A one health and spatiotemporal analysis for targeted intervention

**DOI:** 10.1371/journal.pntd.0014271

**Published:** 2026-04-28

**Authors:** Ping Zhang, Xiaojuan Ma, Ting Pan, Jingxia Dang, Dongfeng Pan, Mingbo Chen, Peifeng Liang

There are errors in the author affiliations. The correct affiliations are as follows:

Ping Zhang¹^,^³, Xiaojuan Ma¹^,^³, Ting Pan¹^,^³, Jingxia Dang¹^,^³, Dongfeng Pan⁴, Mingbo Chen¹^,^³, Peifeng Liang²

**1** School of Public Health, Ningxia Medical University, Yinchuan, China, **2** Department of Medical Affairs, People’s Hospital of Ningxia Hui Autonomous Region, Ningxia Medical University, Yinchuan, China, **3** Ningxia Key Laboratory of Environmental Factors and Chronic Disease Control, Yinchuan, China, **4** Department of Emergency Medicine, People’s Hospital of Ningxia Hui Autonomous Region, Ningxia Medical University, Yinchuan, China
