# Supplementary figures and images for: Socioeconomic drivers of human Brucellosis in Ningxia, China: A one health and spatiotemporal analysis for targeted intervention
Source: PLoS Negl Trop Dis. 2026 Mar 16;20(3):e0014124. doi: 10.1371/journal.pntd.0014124 (PMC13020972; doi:10.1371/journal.pntd.0014124)

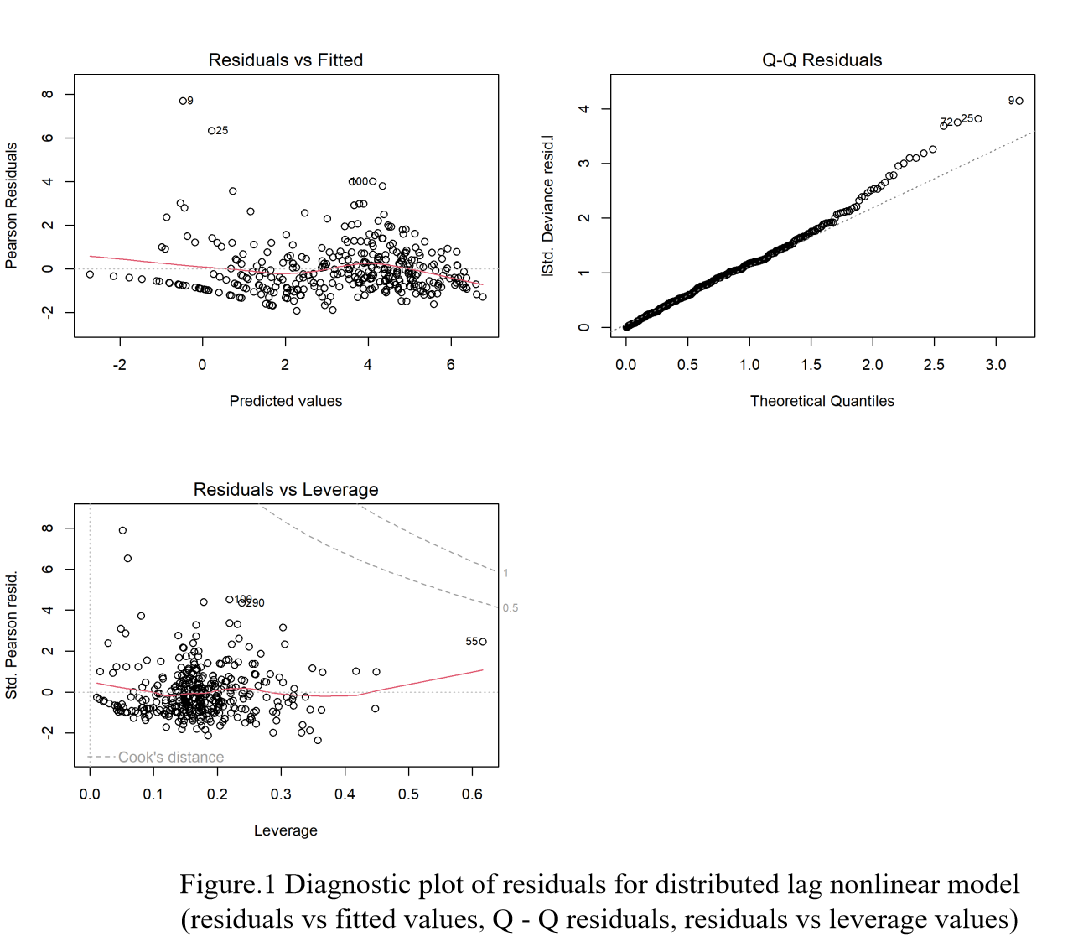

Supplement: S1 Fig — Four-panel diagnostic plot of the negative binomial DLNM residuals: (A) residuals versus fitted values, (B) Q-Q plot of residuals, (C) scale-location plot, and (D) residuals versus leverage values with Cook‘s distance contours. These plots were used to validate key model assumptions including homogeneity of variance, distributional adequacy, and to identify potential influential observations. (TIF) [file pntd.0014124.s001.tif]
